# Film as Ethical Experience in Engineering Education: Three Modes of Ethical Engagement

**DOI:** 10.1007/s11948-026-00636-6

**Published:** 2026-10-16

**Authors:** Nikolas Käkelä, Thomas Taro Lennerfors, Sarah Hitt

**Affiliations:** 1 School of Engineering, Jönköping University, Jönköping, Sweden; 2 Division of Industrial Engineering and Management, Uppsala University, Uppsala, Sweden; 3 New Model Institute for Technology and Engineering, Hereford, United Kingdom of Great Britain and Northern Ireland

**Keywords:** Engineering ethics education, Film pedagogy, Cinematic ethics, Moral imagination, Arts-based learning, Ethical engagement

## Abstract

Engineering ethics education has often been criticized for relying heavily on abstract principles, professional codes, and decontextualized case studies, which can limit students’ emotional and imaginative engagement with ethical issues. This study examines how engineering students articulate different aspects of ethical engagement after participating in a scaffolded film-based teaching intervention. The intervention was implemented in a master’s-level course at a Swedish university, where students watched Hayao Miyazaki’s *The Wind Rises* (2013) and produced written reflections on ethical issues raised by the film. The analysis draws on 76 student reflections and follows a qualitative, abductive approach presented using a Gioia-inspired data structure. Theoretically, the study combines perspectives from engineering ethics education with the concept of cinematic ethics, which emphasizes identification with fictional characters, reflection on moral emotions, and the aesthetic expansion of moral imagination. The findings reveal three interwoven modes of ethical engagement through which students interpreted the film: professional responsibility, identification and moral emotion, and aesthetic mediation of ethical imagination. The study highlights these interwoven modes of ethical engagement, stressing the need for engineering ethics education to be aware of, respond to, and utilize different modes, including aesthetic mediation. Furthermore, it reflects on learner positions in arts-based pedagogies, stressing the potential of the viewer position for stimulating ethical reflection. Finally, it reminds educators of the importance of a scaffolded pedagogical setting for film-based pedagogy.

## Introduction

Engineering ethics education has long aimed to cultivate students’ moral awareness, judgment, and professional responsibility (Herkert, 2000; Rest, 1982; Lennerfors et al., 2020; Tormey et al., 2015), yet concerns remain that prevailing pedagogical approaches rely too heavily on abstract principles, professional codes, and decontextualized case studies, resulting in engagement with ethical situations that can be described as “thin,” meaning that they tend to be simplistic, require minimal emotional investment, and not encourage in-depth reflection (Walling, 2015; Mitcham, 2009; Roeser, 2012). Scholarship in ethics education emphasizes the importance of engaging students emotionally, situating ethical reflection within complex social contexts, and supporting the development of moral judgment through experiential and narrative forms of learning (Sunderland, 2019; Voss, 2013; Mitcham, 2009; Morrison, 2020; Snieder & Zhu, 2020; Zhu et al., 2025). Within this broader educational landscape, film has received attention as a medium capable of presenting “thick” ethical situations that engage viewers emotionally and cognitively, thereby supporting reflective and morally meaningful learning experiences.

Research on cinematic ethics suggests that film can function not merely as a vehicle for illustrating ethical problems, but as a medium of ethical experience in its own right (Sinnerbrink, 2019; Hitt & Lennerfors, 2022; MacAllister, 2023). By inviting emotional engagement with fictional characters, encouraging reflection on one’s own moral responses, and challenging viewers’ assumptions through aesthetic form, film can contribute to learning about ethics. While film pedagogy has been examined in fields such as medical and business ethics, the systematic use of film in engineering ethics education remains underexplored (Berne, 2001; Hess & Fore, 2018), despite the central role of engineering in shaping the social and technological futures so often depicted cinematically (Hitt & Lennerfors, 2022). Not least, empirical studies investigating how engineering students become engaged in ethical issues through cinematic experience remain limited.

The present study addresses this gap by examining students’ responses to a film-based engineering ethics intervention. Drawing on qualitative analysis of written reflections following a screening of *The Wind Rises* (Miyazaki, 2013), the study explores how students engage with ethical issues related to the cinematic experience, which we term “ethical engagement” throughout this article. Through this empirically grounded approach, the paper both explores how ethical engagement is constructed, and offers guidance for educators interested in enriching their approach to engineering ethics education. Against this background, the study asks:

### RQ

How do engineering students articulate different aspects of ethical engagement after viewing and reflecting on a film in an engineering ethics module?

By empirically examining how ethical engagement is constructed within a pedagogically scaffolded setting, the study contributes to ongoing discussions about experiential and arts-based approaches in engineering ethics education.

## Theoretical Framing

### Engineering Ethics Education

Researchers broadly agree that ethics education should aim to improve students’ sensitivity to ethical issues, increase student knowledge of relevant standards, improve ethical judgment, and increase ethical will-power (Herkert, 2000; Rest, 1982; Lennerfors et al., 2020; Tormey et al., 2015). Despite this agreement, critics note that ethics education often focuses too narrowly on professional codes and workplace cases, neglecting emotion, moral sensitivity, and broader social contexts that imbue ethical issues (Walling, 2015; Zhu et al., 2025; Kotluk & Tormey, 2025). This can lead to an overly individualistic and cognitive pedagogical emphasis, where ethical frameworks are treated as abstract exercises rather than guides for real-world action (Mitcham, 2009; Roeser, 2012; Claussen et al., 2026).

While codes and moral reasoning remain important, there has been an ongoing critique within the literature that calls for pedagogical approaches that connect ethical reflection to lived experience, social and ecological context, personal values, and professional responsibility (Sunderland, 2019; Voss, 2013; Morrison, 2020; Snieder & Zhu, 2020; Zhu et al., 2025). Similar concerns are reflected in reviews of engineering ethics education, which point to the need for more experiential, dialogical, and reflective approaches (Hess & Fore, 2018; Polmear et al., 2025; Martin, 2025). Another stream of literature points to the importance of considering emotions in engineering ethics education, stressing the inherent connections between the emotional and the cognitive (see Tormey et al., 2025; Kotluk et al., 2025).

For the present study, this literature provides the educational problem to which film-based pedagogy responds: how to create conditions in which students encounter ethical issues not only as abstract problems, but as emotionally charged, socially embedded, and interpretively complex situations.

### Film Pedagogy and Ethics Education

Film has been used pedagogically since at least the 1970s (Wegner, 1977) and is well established in business and medical ethics education, where it has been used to support critical thinking, emotional awareness, virtue development, and consideration of stakeholder perspectives (Searight & Allmayer, 2014; Skorin-Kapov & Benson, 2018). In engineering ethics education, however, film remains comparatively underdeveloped. Existing work tends either to mention film only briefly, to focus on purpose-made educational films such as *Henry’s Daughters or Incident at Morales*, or to examine problematic consequences of engineering-related situations (Berne, 2001; Loui, 2006; Riley, 2013; Hess & Fore, 2018).

Purpose-made educational films can address ethical content explicitly and support learning outcomes (Itani, 2013; Loui, 2006), but they may also appear didactic and lack the narrative and aesthetic complexity of commercial films (Wegner, 1977). Hitt and Lennerfors (2022) argue that Miyazaki’s *The Wind Rises* can serve as an exemplar for engineering ethics education because it brings up ethical issues connected to technological development in turbulent times, touches upon the role of agency in large scale technological systems, and does so in aesthetically compelling ways. However, empirical studies of how engineering students respond to such cinematic material remain scarce.

### Cinema as a Medium of Ethical Experience

To conceptualize how film can contribute to engineering ethics education, this study, as in Hitt and Lennerfors (2022), draws on the work of Robert Sinnerbrink, who argues that cinema should be understood not merely as a vehicle for representing ethical problems, but as a medium of ethical experience in its own right. He distinguishes this view from approaches that focus primarily on ethics *in* cinema – the narrative content of the film, ethics of cinematic representation – including whether the film is truthful to what it depicts, or ethics of cinema *as* ideology – an unmasking of the underlying values of the film. What these approaches tend to overlook, according to Sinnerbrink, is cinema’s capacity to deepen the understanding of moral situations because of the complex forms of emotional engagement it offers (Sinnerbrink, 2019).

Sinnerbrink explains that film promotes ethical experience in three ways. First, viewers engage with fictional characters, their actions, viewpoints, and lived situations. Such engagement can bring out the complexity of ethical decision-making by placing characters within interpersonal, social, and political contexts. Film can therefore present ethically “thick” situations that invite reflection on judgment and action under complex circumstances.

Second, going beyond identification or rejection of these characters, viewers are prompted to reflect on their own emotions, and feelings of moral approval or disapproval. This reflective stance enables viewers to step back from the narrative and consider whether the characters acted rightly or wrongly, or how they themselves might have acted in similar situations.

Third, ethical experience is also generated through the aesthetics of cinematic experience, which can expand viewers’ moral perspectives. Sinnerbrink (2018) argues that “spectators may reach a deeper understanding of moral situations by having their assumptions or beliefs challenged via complex forms of emotional engagement coupled with critical reflection arising from this ethical experience” (p. 199). Through aesthetic mediation, film can thus cultivate moral imagination: the capacity to envision alternative possibilities, futures, and forms of responsibility beyond immediate judgement.

In this study, these three aspects of cinematic ethical experience informed the design of the film-based intervention and, in particular, the orientation of the written reflection prompts.

### Reflection, Dialogue, and Pedagogical Scaffolding

The ethical potential of film depends on how cinematic experience is pedagogically framed. Scholarship on engineering ethics teaching emphasizes reflection, dialogue, case-based reasoning, perspective-taking, and the creation of written or artistic artefacts as important ways of supporting ethical engagement (Polmear et al., 2025; Martin, 2025; Andersson & Brodin, 2026). Dialogue has been described as central to ethical reflection (Marin et al., 2025), and film discussion has been used in medical ethics education to promote ethical thinking and moral reasoning (Self et al., 1993; Green et al., 1995). Hitt et al. (2025) similarly argue that guided interpretation of narrative and artistic experiences can help students relate such experiences to engineering practice.

This is important for the present study because the film was not used as a standalone intervention. Students first encountered an engineering ethics framework, then watched *The Wind Rises* (2013), wrote individual reflections, and participated in seminar dialogue. The study therefore examines a scaffolded cinematic ethics intervention, where film functions as a shared narrative and aesthetic reference point around which reflection and dialogue are organized.

## Research Method

### Research Context and Pedagogical Design

This study was conducted within a master’s-level course on Research Methodology at a Swedish university in 2025. The course was taught in English and jointly attended by students from four master’s-level programs with diverse engineering specializations, including logistics and supply chain management, production engineering, and product design. As part of the course, students participated in a dedicated module on engineering ethics, designed to introduce ethical reasoning and professional responsibility within the broader context of engineering practice. For most students, this represented their first substantial exposure to engineering ethics as a formal subject. This assessment was based on the instructors’ familiarity with the participating programs and their curricula rather than on a separate student survey. While some students may have encountered ethics during their bachelor’s-level studies, few had previously engaged in structured reflection or discussion of ethical dilemmas in engineering.

The pedagogical sequence of the engineering ethics module followed a lecture-film-reflection-seminar design. First, a lecture introduced foundational ideas in engineering ethics, drawing on Lennerfors’ (2019) approach to present ethics as a process-oriented practice encompassing awareness, responsibility, critical thinking, and action (see Sect.  3.3). Ethics was addressed both descriptively and normatively, emphasizing how engineers may reflect on what is right or good while navigating value pluralism, uncertainty, and trade-offs in practice.

Second, students watched *The Wind Rises* (2013), directed by Hayao Miyazaki. The film is an animated historical drama loosely inspired by the life of Jiro Horikoshi, the aeronautical engineer associated with the Mitsubishi Zero fighter aircraft. It portrays Jiro’s will to create beautiful aircraft against the backdrop of war and destruction. The film was therefore selected because it foregrounds ethical tensions relevant to engineering work, including innovation, professional identity, dual-use technologies, and responsibility for consequences. The screening took place in a cinema setting; students who were unable to attend were instructed to watch the film individually prior to completing the subsequent reflection assignment.

Following the film, students were instructed to write a 200–300-word reflection. The assignment prompted students to (1) identify an ethical dilemma or question presented in the film that related to the role of engineers in society, (2) state whether they agreed with Jiro Horikoshi’s actions and moral values and explain why, (3) identify a scene that provided an interesting representation of ethics in engineering or technology development and explain why they selected it, and (4) formulate an open-ended question for discussion. The purpose of the written reflection was not to assess students’ knowledge of ethical theories, but to elicit engagement with ethical issues in engineering.

Finally, students participated in a seminar intended to deepen ethical reflection through dialogue. The class was divided into three parallel seminar sessions, each further organized into small groups of five to six students. Discussions were structured around the dilemmas and questions raised in the written reflections, and each seminar concluded with a plenary discussion facilitated by instructors. While these seminar discussions formed an integral part of the pedagogical design, they are not treated as empirical material in the present analysis. They formed part of the broader pedagogical sequence and gave the written assignment a dialogical purpose: students wrote in preparation for subsequent discussion. The written reflections therefore constitute the sole empirical material analysed in this study.

### Participants and Data Collection

In total, 80 students participated in the module, representing a range of engineering backgrounds and nationalities. Completion of the written reflection and participation in the seminar were compulsory components of the course. Students were informed in writing that their responses would also be used for the present research study, and that they could opt out without consequences for course participation or assessment. No personal or demographic identifiers were collected beyond program affiliation, and all reflections were anonymized prior to analysis. Four students opted to have their reflections excluded, resulting in a final dataset of 76 reflections. Under the Swedish Ethical Review Act (2003:460, §§ 3–4), the study did not fall within the categories of research requiring formal ethical review, and ethical approval was therefore not required.

### Theoretical Foundations of the Intervention

The pedagogical intervention was informed by two complementary theoretical perspectives: Lennerfors’ (2019) framework for engineering ethics and Sinnerbrink’s (2018, 2019) account of cinematic ethics. The two perspectives informed different elements of the intervention. Lennerfors primarily shaped the engineering ethics lecture preceding the film screening, while Sinnerbrink informed the cinematic orientation of the written reflection prompts.

Lennerfors’ approach to engineering ethics education, materialized in the book *Ethics in Engineering* (2019), was used as a starting point for the engineering ethics component of the intervention. It builds on five ideas that together are designed to alleviate some of the problems related to engineering education mentioned in the introduction, such as an individualistic and cognitive focus (Lennerfors et al., 2020). First, engineering ethics is framed as problem formulation and problem solving, which is seen as crucial to engineering practice. Problem formulation and problem solving are organized around four concepts: awareness, responsibility, critical thinking, and action. *Awareness* concerns sensitivity to the ways engineers affect the world through their work with technology, their relationships with others, and the interaction between professional and private life. *Responsibility* concerns both forward- and backward-looking responsibility and directs attention to the relationship between an engineer’s agency, the constraints under which the engineer acts, and the impact of engineering activity. *Critical thinking* concerns deliberation over possible courses of action, including consideration of consequences, duties, rights, virtues, relationships, and broader socio-ecological implications. *Action* emphasizes that engineering ethics is action oriented and includes the capacity and willingness to act on the outcome of the critical thinking process.

Second, the approach has a range of interdisciplinary sources of inspiration, including normative ethics, economics, psychology, and sociology, and ethical theories from different parts of the world, acknowledging the open and interdisciplinary character of engineering ethics education (see Chance et al., 2025, Sect.  2). Third, it builds on both positive and negative emotional registers, acknowledging the emotional character of engineering work, thereby eschewing engineering ethics as the study of avoiding engineering disasters by also focusing on the positive potential of engineering. Fourth, the approach highlights everyday life rather than extraordinary situations, in other words, paying close attention to the handling of ethical issues that students are likely to or might already have experienced in their lives. Fifth, the approach stresses the engagement of both cognitive and emotional registers of the student to stimulate attention and motivation, including the mobilization of art, which is in line with this paper’s focus on ethical engagement.

More practically, the introductory lecture concerned the interdisciplinary nature of engineering ethics and explained that ethical concepts are constructed and subject to reflection and debate. Thereafter the lecture was scaffolded around the ethical process, i.e. awareness, responsibility, critical thinking, and action. Awareness was exemplified by artistic examples such as an analysis of Peter Bruegel the elder’s “Landscape with The Fall of Icarus”, and analyses of the moral implications of technologies such as hammers, hotel keys, benches, and implantable devices. Responsibility was exemplified by discussing a bridge collapse in Stockholm, but also an analysis of Star Wars character Galen Erso’s actions from the viewpoint of professional responsibility. Critical thinking was presented including normative ethical theories, applied to self-driving cars and social robots, and action was discussed as well as the obstacles to ethical action. The lecture ended with problematizations of the presented approach.

While the pedagogical approach by Lennerfors naturally includes film and other art in its pedagogical repertoire, Sinnerbrink’s account of cinematic ethics provided a perspective on the specifically cinematic forms through which ethical issues may be encountered. As discussed in Sect.  2.3, it informed the cinematic orientation of the written reflection prompts, which directed students toward moral evaluation of the protagonist and attention to how ethical issues in engineering and technology development were represented in particular scenes. Contrasting Lennerfors and Sinnerbrink, Lennerfors’ approach is broader and does not say anything specific about film. However, it does build on the importance of emotional engagement, which is in line with Sinnerbrink’s ideas of films being a suitable site for reflecting on moral emotions and judgment. Furthermore, Sinnerbrink’s framework is explicit on the aesthetic mediation of ethical experience, which Lennerfors’ approach is silent about, despite the references to using art for engineering ethics education. Furthermore, Lennerfors’ approach focuses on the agent perspective, rather than Sinnerbrink’s approach of identifying with and reflecting on other characters who engage in ethical action. However, this does not make the approaches incompatible. They reflect, in part, the different positions involved: Lennerfors focuses primarily on engineers as ethical agents, whereas Sinnerbrink considers the ethical experience of viewers engaging with the actions of others through film.

**Fig. 1:**
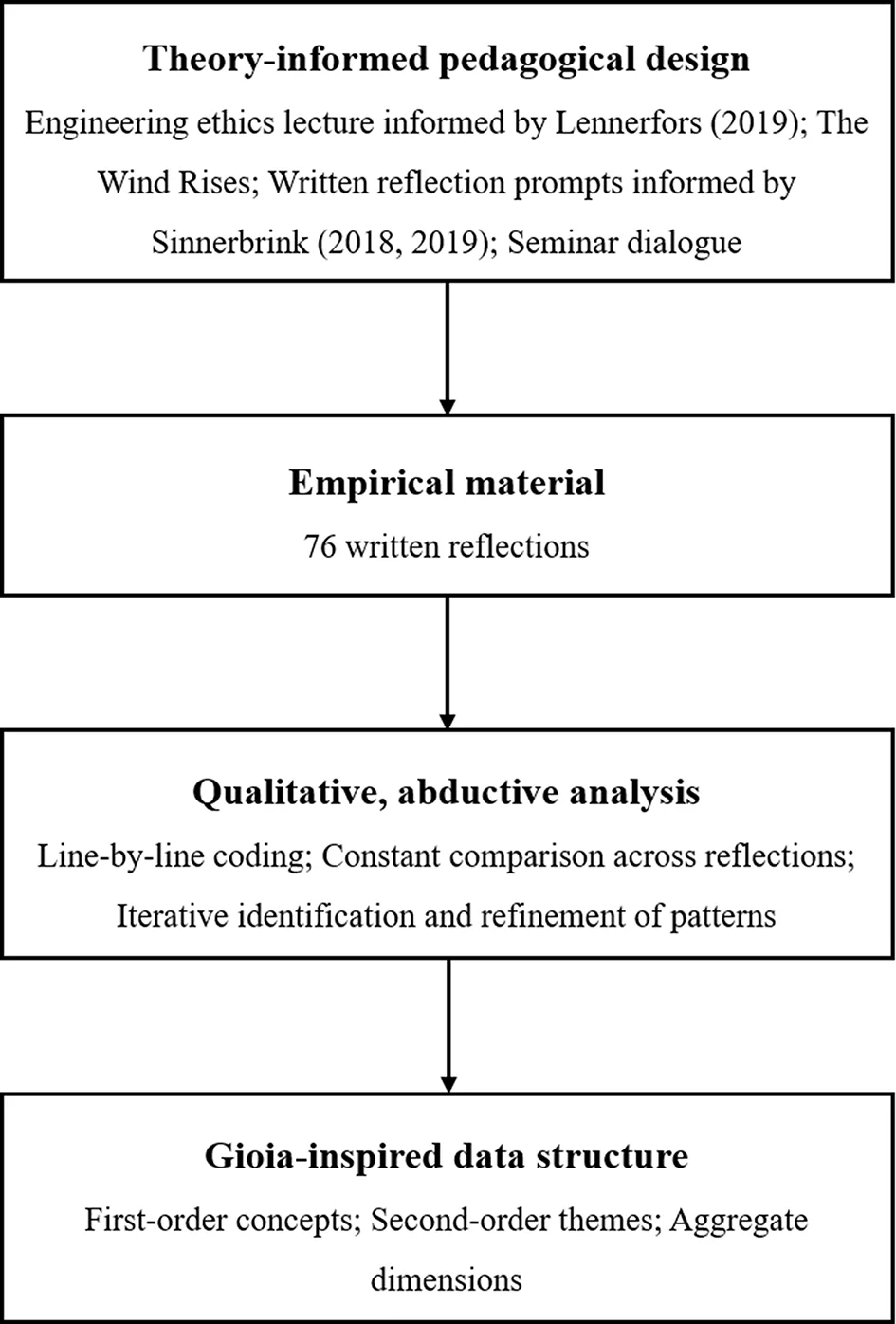
Relationship between the theory-informed pedagogical design, empirical material, and analytical process

The two perspectives therefore informed different elements of the pedagogical design and, consequently, the context in which the empirical material was produced. Neither perspective was subsequently applied as a coding framework in the qualitative analysis. Figure 1 summarizes the relationship between the theory-informed pedagogical design, empirical material, and analytical process.

### Data Processing and Analysis

All written reflections were downloaded from the university’s learning management system and compiled into a single dataset. Each reflection was assigned a pseudonymous identifier (e.g., S01–S76) prior to analysis. The dataset was stored and organized in an Excel workbook, which served as a coding matrix during analysis. The analysis followed a qualitative and abductive approach, presented using a Gioia-inspired data structure (Gioia et al., 2013). The Gioia-inspired format was used to make transparent the progression from student-centred first-order concepts to researcher-developed second-order themes and, ultimately, aggregate dimensions. Coding and category development proceeded iteratively through close reading, comparison across reflections, and refinement of recurring patterns in the material. Throughout the analysis, the terms first-order concepts, second-order themes, and aggregate dimensions refer specifically to these three levels of the data structure. In the Discussion, the three aggregate dimensions are interpreted as modes of ethical engagement, referring to distinct but potentially interrelated ways in which students articulated ethical meaning.

Each reflection was read carefully and coded line by line. Phrases expressing ethical positions, questions, or emotional responses were identified as coded excerpts, staying as close as possible to students’ own language (e.g., “Should engineers refuse harmful work?” or “He just wanted to build something beautiful”). This initial coding generated over 700 coded excerpts, each traceable to its original student identifier. Only minor linguistic adjustments were made to improve clarity without altering meaning. As coding progressed, recurring patterns became apparent. Some statements emphasized professional responsibility, others foregrounded emotional identification with the protagonist, and others focused on aesthetic elements such as beauty, symbolism, and imagination. These patterns also reflected, in part, the pedagogical framing of the reflection assignment, which directed students toward ethical dilemmas, their own positions, ethically significant scenes, and questions for further discussion. This correspondence between the pedagogical design and the resulting patterns was treated as an analytical condition rather than as evidence that the categories emerged independently of the pedagogical intervention. The analytical task was therefore to examine how students articulated ethical engagement, how these articulations varied across the dataset, and how different forms of ethical engagement were connected within individual reflections.

Through comparison of these excerpts, recurring first-order concepts were identified and subsequently grouped into 15 s-order themes, which were organized into three aggregate dimensions. For example, within the aggregate dimension of Professional Responsibility, second-order themes included “Boundaries of professional responsibility” and “Institutional and wartime constraints on agency.” Each theme was supported by multiple reflections to ensure that it represented collective patterns of reasoning rather than isolated viewpoints. To strengthen analytical rigor, coded excerpts were compared across reflections, and themes were retained only when supported by multiple student responses. Representative quotations were selected to illustrate both recurring patterns and variation within the dataset, including agreement, critique, ambivalence, and uncertainty.

Throughout the analysis, constant comparison across reflections was used to refine the developing concepts and themes and maintain their grounding in students’ expressions. The resulting data structure illustrates how students engaged with ethical issues through viewing and reflecting on *The Wind Rises*, articulating dilemmas of professional responsibility, emotional response, and ethical imagination. To preserve readability and visual clarity, each second-order theme in the Gioia data structure is illustrated with two to four representative quotations (see Appendices A, B, and C). These quotations are not exhaustive but exemplify broader patterns within the dataset.

The authors’ role as educators and analysts also shaped the study. The pedagogical intervention was designed and delivered by members of the author team, which provided contextual familiarity with the course, assignment, and seminar format. At the same time, this positioning required reflexive caution. We therefore interpret the reflections as evidence of students’ articulated ethical engagement within a scaffolded educational setting, rather than as direct evidence of changes in ethical competence or learning outcomes.

## Findings

The analysis produced three aggregate dimensions: *Professional responsibility*, *Identification and moral emotion*, and *Aesthetic mediation of ethical imagination*. Table 1 shows a condensed data structure, illustrating how selected student quotes were gradually abstracted into second-order themes and aggregate dimensions. While the analysis generated fifteen second-order themes across the three aggregate dimensions, the presentation further below focuses on the most representative themes within each dimension. These were selected for their conceptual richness and their ability to illustrate the broader patterns of ethical engagement observed in the data. A complete overview of all themes and exemplar quotes is provided in Appendix A, B, and C.

**Table 1:**
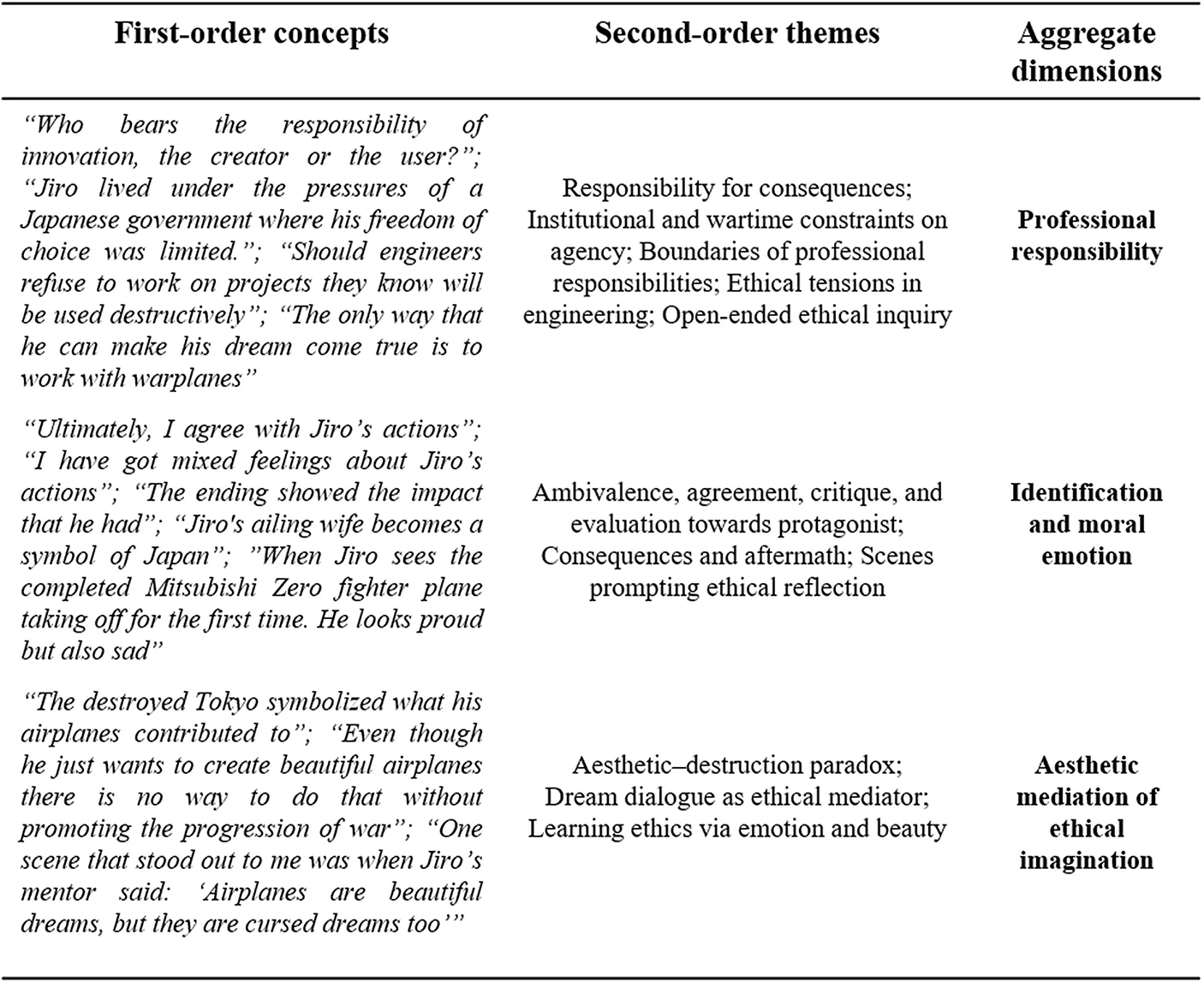
Condensed Gioia data structure showing how student reflections were abstracted into second order themes and aggregate dimensions

### Professional Responsibility

The first dimension captures students’ reflections on moral responsibility within the engineering profession. The themes highlighted below illustrate the progression from contextual constraint to reflective agency that characterized this dimension. Many began by situating Jiro’s actions within his historical and institutional context, describing how social, political, and organizational forces constrained his freedom to act as an individual. This gave rise to the theme *Institutional and wartime pressure constraining agency*, where students considered the extent to which engineers can or cannot resist unethical demands in hierarchical systems. One student wrote, “*I recognize that Jiro lived under the pressures of a Japanese government where his freedom of choice was limited*”. Others drew parallels to contemporary industries, asking whether modern engineers can meaningfully avoid contributing to technologies that can be viewed as harmful.

A second theme, *Ethical tensions in engineering*, captured how students reflected on the moral tensions embedded in everyday engineering decisions. Several referred to scenes in which Jiro proposed removing the weapons from his aircraft design to reduce weight, which drew laughter from his colleagues. Others pointed to his admiration for German aeronautical advances despite their militaristic purpose. These reflections show how students recognized the difficulty of aligning technical ingenuity with ethical responsibility. As one student summarized, *“It shows the struggle of time*,* money*,* and safety when you work as an engineer.”* As students grappled with such tensions, some began to move beyond description toward explicit moral inquiry. For example, one student first reflected on how Jiro’s work was shaped by wartime demands and professional expectations, noting that “*he didn’t really have a choice if he wanted to continue working as an engineer*,” before posing the question: “*Should engineers refuse to work on projects they know will be used destructively*,* even if it means sacrificing their careers?*” Under the theme *Open-ended ethical inquiry*, students formulated genuine questions rather than firm judgments about professional duty. Such questions reflected a process of moral deliberation rather than the application of rules for ethical action.

Together, these themes show how students articulated *professional responsibility* as relational and context-dependent, shaped by social, organizational, and historical constraints as well as by questions of individual agency.

### Identification and Moral Emotion

The second dimension centers on how students identified and engaged emotionally with the protagonist, by positioning themselves in relation to Jiro with varying degrees of empathy, agreement, critique, and unease. The theme *Agreement with protagonist* illustrates how students admired Jiro’s devotion to craftsmanship and innovation, even while recognizing its moral ambiguity. For example, one student wrote, *“It may have been his only way to pursue his dream*,* and someone else would have designed military planes regardless.”* Others described how they could understand his choices, but not necessarily agree with them. Thus, the responses did not converge on a single moral evaluation of Jiro. Some students expressed sympathy or agreement, others criticized his detachment from consequences, and many occupied an ambivalent position between admiration and unease. This ambivalence is illustrated by one student’s reflection: *“I respect Jiro’s passion and commitment*,* but I don’t fully agree with his choice to ignore the consequences.”* The theme *Scene prompting ethical reflection* was linked to the film’s emotionally charged moments, particularly those combining personal intimacy with professional duty. Scenes of Jiro working beside his dying wife were repeatedly cited as moments of cognitive and emotional dissonance. As one student reflected, *“He’s loving and caring when the suffering is personal to him*,* but at the same time putting energy into something that will make others suffer.”*

Together, these themes show how students articulated ethical reflection through *identification* with the protagonist and through *moral emotions* such as empathy, admiration, discomfort, and ambivalence.

### Aesthetic Mediation of Ethical Imagination

The third and final dimension reflects how students engaged with the film’s aesthetic and symbolic layers to explore ethical imagination. Here, ethical understanding was not only cognitive or emotional, but also aesthetic, mediated by beauty, symbolism, and metaphor. Central to this dimension was the theme *Dream dialogue as ethical mediator*. Many students interpreted the recurring dream sequences between Jiro and the Italian engineer Caproni as a way of exploring the moral cost of creation, including the question of whether one would prefer “*a world with pyramids or without them*”. A related theme, *Aesthetic–destruction paradox*, captured how students reflected on the duality of beauty and destruction in engineering. One student commented: “*The destroyed Tokyo symbolized what his airplanes contributed to*,* even though his original dream was to create something beautiful.*” Another student commented: “*In the scene when Jiro meets Caproni in his dreams*,* Caproni asks whether designing beautiful planes is worth the destruction they cause. The animation contrasts grey tones representing destruction with the colourful tones of Jiro’s dream*,* emphasizing the moral weight of his engineering decisions and the tension between creativity and consequence.*” This awareness transcended simple moral judgment, enabling students to see ethics through imagination and symbolism.

Together, these themes illustrate how students used aesthetic and symbolic features of the film to articulate *ethical imagination* and to make sense of moral complexity through beauty, destruction, and metaphor.

### Interweaving of Modes within Individual Reflections

Although the three aggregate dimensions were analytically distinguished, they were often interconnected within individual reflections. Of the 76 reflections, 63 contained coded excerpts associated with more than one aggregate dimension, and 16 contained excerpts associated with all three. Because the reflection assignment contained four prompts addressing different aspects of the film and ethical reflection, some co-occurrence at the level of the full reflection may partly reflect the structure of the assignment. That students did not necessarily engage with professional responsibility, moral emotion, and aesthetic imagination as separate forms of ethical reflection. The interweaving was also visible within individual responses to the assignment prompts. For example, while reflecting on a dream sequence between Jiro and Caproni, one student described the scene as illustrating the difference between how something is created and how it is ultimately used. The student characterized this tension as “ironic and sad” before asking whether engineers can really decide what happens to the things they invent. Within a single response, aesthetic interpretation of the dream sequence was thus connected to emotional evaluation and to a question of professional responsibility. This example illustrates how the modes could operate together within a single process of ethical meaning-making. The distinction between the three aggregate dimensions is therefore analytical rather than indicative of three mutually exclusive forms of engagement. These patterns describe how students articulated ethical meaning within the scaffolded intervention and should not be interpreted as evidence that the film independently produced these forms of engagement.

## Discussion

The findings highlight three interrelated modes of ethical engagement: ethics as situated professional practice, ethical sensitivity through identification and moral emotion, and the aesthetic mediation of moral imagination. Rather than discussing each mode separately, this section focuses on how these modes were interwoven within students’ reflections, how film positions learners in relation to other arts-based pedagogies, and how pedagogical scaffolding shaped the forms of ethical engagement observed.

### Ethical Engagement as An Interwoven Process

The three modes of engagement illustrate ethical engagement as an integrated and multimodal process. The findings provide insight into the structure of ethical engagement in a narratively and aesthetically mediated learning environment. Students did not engage with ethical issues solely through professional reasoning, nor solely through emotional response or aesthetic interpretation. Instead, these modes were interwoven within individual reflections. This suggests that ethical meaning-making may involve movement between professional, emotional, and aesthetic modes rather than engagement through these forms in isolation.

Students’ reflections consistently framed ethical responsibility as something negotiated within historically, organizationally, and institutionally constrained contexts. Students engaged with responsibility as relational, contingent, and shaped by power structures, hierarchy, and socio-political pressures, rather than treating ethical action as a matter of applying abstract rules or professional codes. This orientation aligns closely with core aims of engineering ethics education that emphasize responsibility, ethical judgment, and contextual awareness (Herkert, 2000; Lennerfors et al., 2020; Tormey et al., 2015, Zhu et al., 2025), while also resonating with long-standing critiques of overly individualistic and decontextualized approaches to ethics instruction (Mitcham, 2009; Roeser, 2012; Hitt & Lennerfors, 2022). What the present findings add is an empirical illustration of how students articulated responsibility as situated and politically shaped, by being drawn into an experiential narrative where institutional constraint is felt and discussed rather than merely described. Importantly, however, these reflections did not remain confined to questions of professional responsibility.

The findings are consistent with claims in the ethics education literature that emotion plays a constitutive role in ethical reflection and ethical sensemaking (Roeser, 2012; Snieder & Zhu, 2020, Tormey et al., 2025), and that there is an interweaving of the cognitive and the emotional. Through cinematic identification, students encountered ethically complex situations that resisted simple moral categorization. Emotional responses appeared to provoke reflection by prompting students to interrogate their own values, assumptions, and boundaries of responsibility. This pattern aligns with Sinnerbrink’s (2019) account of cinematic ethics, in which ethical experience emerges through emotional engagement with fictional characters and their circumstances. Notably, students’ frequent ambivalence toward the protagonist suggests that emotional identification can coexist with moral critique.

Beyond cognitive reasoning and moral emotion, students’ reflections also demonstrated how aesthetic features of the film mediated ethical imagination. References to symbolism, visual contrasts, narrative metaphors, and dream sequences indicate that ethical understanding was shaped through imaginative engagement with alternative possibilities, futures, and consequences associated with technological progress as depicted aesthetically. This mode of engagement corresponds closely to discussions of moral imagination in ethics education (Callahan, 1980; Lennerfors et al., 2020) and to Sinnerbrink’s (2019) account of aesthetic broadening of moral perspective. While moral imagination is frequently discussed as a desirable learning outcome, it is less commonly examined empirically in engineering ethics education compared with other outcomes emphasized in review work (Hess & Fore, 2018; Polmear et al., 2025). The findings indicate that students used cinematic aesthetics as a resource for imaginative ethical reasoning, allowing them to engage with moral complexity through symbolism and visual storytelling rather than propositional argument alone. This pattern resonates with holistic models of ethics education that emphasize the interdependence of awareness, responsibility, critical thinking, and action (Lennerfors, 2019). Although the role of emotion has now become a crucial component within discussions about engineering ethics education, we find that the aesthetic dimension is still often downplayed in such education. Similar to how Kotluk and Tormey (2025) have stressed the emotional component of case studies that are often seen as being focused on the cognitive, it is important to study the aesthetic dimensions in pedagogical practice that go beyond arts-based pedagogies, understanding more pedagogical situations as holistic experiences, where students can shift between different modes of ethical engagement.

### The Importance of Learner Position in Arts-Based Education

Another area of research to which this study makes a contribution is the consideration of the learner position during the aesthetic experience in arts-based pedagogies. For instance, some scholarship focuses on learners *doing* the making or performing of art such as dance (Foster & Ojanen, 2025), drama (Coleman & Dick, 2016), or narrative and visual art (Yi et al., 2025). Others consider *viewing* or *reviewing* arts-focused experiences through interpretation and reflection, such as reading fiction (Andersson & Brodin, 2026), visiting a museum (Delany & Gaunt, 2018), or watching film (Calabor et al., 2025). This seems like a critical difference; yet these examples from multiple disciplines and educational environments suggest that even when students are spectators (viewers) rather than participants (doers), ethical reflection still emerges through interpretation of a shared experience.

A further nuance emerges in the case of the cinematic experience, as some philosophers of film argue that the immersive and shared experience of viewing a film alongside others creates a different level of experience, or a “filmworld” that elicits an “existential-ethical potential” in viewers (de Roo, 2019). This is also alluded to in work such as Manolis et al. (2025) who describe an example where a film was shown to students who were themselves based in the location of the events depicted, heightening the personal emotional relationship to the ethical issue. A related experience could be that of engaging with AR/VR to explore ethical concepts (Starke et al., 2026).

Ultimately, we can see that arts-based approaches place learners in different positions to the ethical situations being explored. For instance, if they are assuming a role in a drama, they may engage differently with the situation than if they are simply watching the drama play out, because they may on some level adopt the position of the character, or conversely reject having to adopt it. It is therefore possible that these positions could affect the ethical experience that they have, but further research is needed to explore this concept. However, the studies referenced here which reflect a variety of arts-based approaches all posit that art and aesthetic experiences can help students engage ethically, through processes like seeing both sides of an issue, practicing introspection, creating meaning, and becoming inspired (Lad & Skinner, 2024).

### Pedagogical Scaffolding and Implications for Engineering Ethics Education

Although this has been repeatedly argued in research on engineering ethics education, we feel that it is still important to stress the pedagogical scaffolding of film-based, as well as other pedagogical, interventions. The findings underscore the importance of pedagogical sequencing. In this study, ethical engagement unfolded in a context where film viewing was combined with structured written reflection and guided seminar discussion. The film did not function as a standalone ethics intervention, but as a common narrative and experiential anchor around which ethical dialogue and deliberation could develop. Seen in this way, the pedagogical value of film lies not solely in the cinematic experience itself, but in how that experience is mobilized through reflection, discussion, and connection to professional practice. Such designs can help address persistent concerns about the “thinness” of ethics education by offering ethically “thick” experiences that complement more traditional instructional methods (Walling, 2015; Mitcham, 2009; Roeser, 2012; Sunderland, 2019; Voss, 2013; Morrison, 2020; Snieder & Zhu, 2020). This interpretation aligns with recent syntheses of engineering ethics teaching methods that emphasize experiential, dialogical, and reflective approaches (Polmear et al., 2025; Martin, 2025; Marin et al., 2025). For engineering ethics education, film can therefore be understood less as an alternative to established pedagogical methods than as one component within a broader pedagogical repertoire. Its contribution lies in providing a common narrative and aesthetic experience that can subsequently be mobilized through individual reflection and collective dialogue.

### Limitations and Future Research

This study is limited by its focus on a single film and a single educational context, as well as by its reliance on written student reflections. Future research could explore how different films, genres, or narrative forms shape ethical engagement; compare film-based pedagogy with other arts-based approaches; or examine the longer-term impact of cinematic ethics education on professional identity formation. Further empirical work could also investigate how specific design features of film-based assignments shape different aspects of ethical engagement. Comparative research could particularly examine whether different learner positions, such as spectatorship and participation, are associated with different forms of ethical reflection. Furthermore, future research could explore the aesthetic dimension of different forms of pedagogies in engineering ethics education.

An additional limitation concerns the close alignment between the pedagogical design, theoretical framing, and analytical categories. Students were explicitly invited to reflect on ethical dilemmas, the protagonist’s actions, and ethically significant scenes, while the pedagogical design and reflection prompts were informed by Sinnerbrink’s account of cinematic ethics, which emphasizes identification, moral emotion, and aesthetic imagination. Consequently, the study cannot disentangle the influence of the film itself from that of the reflection prompts and broader pedagogical scaffolding. Nor can it determine whether similar forms of ethical engagement would emerge in response to alternative instructional approaches.

## Conclusions

This study contributes to engineering ethics education by showing how professional, emotional, and imaginative forms of ethical engagement can become interwoven when students interpret a cinematic narrative within a scaffolded pedagogical setting. The findings identified three interrelated modes of ethical engagement: professional responsibility, identification and moral emotion, and aesthetic mediation of ethical imagination. Their significance lies in how students moved between these modes when making sense of questions concerning engineering agency, professional responsibility, and technological consequences. In this respect, the study provides empirical insight into ethical engagement as an interconnected process rather than a set of separate forms of ethical reasoning. This implies that engineering ethics education could benefit from considering these modes of engagement, when designing pedagogical interventions beyond film-based education. This highlights aesthetic mediation as an additional dimension to consider alongside the emotional and cognitive aspects already emphasized in engineering ethics education.

The study also points to viewership as a distinctive learner position within arts-based ethics education. Film allows students to become emotionally and imaginatively involved in an ethical situation while retaining interpretive distance from the characters and events represented. In the present study, this was reflected in students’ capacity to identify with and admire the protagonist while simultaneously criticizing his decisions and remaining ambivalent about his responsibility. Distinguishing such learner positions may help develop a more differentiated understanding of how different arts-based approaches contribute to engineering ethics education and provide a useful direction for comparative research.

The contribution of the study is therefore not to demonstrate that film, by itself, improves ethical learning. Rather, it is to provide an empirical illustration of how students construct ethical meaning when cinematic experience is combined with structured reflection and dialogue. By integrating perspectives from engineering ethics education and cinematic ethics, the study highlights the potential value of narratively and aesthetically mediated pedagogies for enriching ethical reflection in engineering education. Film should not be understood as a standalone solution to the challenges of ethics education, but as one possible resource through which students can engage with ethical issues as emotionally resonant, contextually situated, and open to interpretation.

## Appendix A

*Gioia data structure for Aggregate Dimension 1 – Professional responsibility.* 

## Appendix B

*Gioia data structure for Aggregate Dimension 2 – Identification and moral emotion.* 

## Appendix C

*Gioia data structure for Aggregate Dimension 3 – Aesthetic mediation of ethical imagination.* 

## Data Availability

The datasets generated and analysed during the current study are not publicly available due to ethical considerations and the presence of student educational data but are available from the corresponding author on reasonable request.
